# *Indigofera oblongifolia* Prevents Lead Acetate-Induced Hepatotoxicity, Oxidative Stress, Fibrosis and Apoptosis in Rats

**DOI:** 10.1371/journal.pone.0158965

**Published:** 2016-07-08

**Authors:** Ahmed E. Abdel Moneim

**Affiliations:** Department of Zoology and Entomology, Faculty of Science, Helwan University, Cairo, Egypt; University of South Alabama Mitchell Cancer Institute, UNITED STATES

## Abstract

The current study was aimed to evaluate the preventive effects of *Indigofera oblongifolia* leaf extract (IOLE) on lead acetate (PbAc)-induced hepatotoxicity in adult male Wistar rats. PbAc was intraperitoneally injected at a dose of 20 mg/kg body weight for 5 days alone or in combination with the IOLE (100 mg/kg). Liver lead concentration and oxidative stress markers such as lipid peroxidation, hydrogen peroxide, nitric oxide, and glutathione content were investigated in addition to the enzymatic antioxidant activities. PbAc injection caused a significant elevation in the liver function parameters, lead level, lipid peroxidation, hydrogen peroxide, and nitric oxide, with a concomitant decline in the glutathione content compared with the control, accompanied by a significant inhibition of antioxidant enzyme activities. The induction of oxidative stress, lead accumulation, and histological alterations in the liver were successfully minimized by pre-administration of IOLE. In addition, the PbAc group showed increase in the levels of Bax, caspase-3, and matrix metalloproteinase-9 proteins, while the expression of Bcl-2 protein was decreased. Prior administration of IOLE significantly mitigated apoptosis and fibrosis in the liver. Finally, the major components in *I*. *oblongifolia* extract were identified as polyphenols, flavonoids, and organic acids using liquid chromatography coupled mass spectroscopy. Thus, the findings of the current study revealed that *I*. *oblongifolia* had protective, anti-fibrotic, antioxidant, and anti-apoptotic activities on PbAc-induced hepatotoxicity. The beneficial effects of *I*. *oblongifolia* were in part mediated by Nrf2/HO-1 pathway.

## Introduction

Lead (Pb) is a toxic pollutant, and occupational as well as environmental exposures remain a global health problem. Continuous exposure to Pb even at low concentrations has been established as a risk factor causing hepatotoxicity, nephrotoxicity, and reproductive and behavioral dysfunctions [1–3]. Among the organs affected by Pb toxicity, the liver is the most common depository of Pb, followed by the kidney [4]. The liver is the first organ exposed to enterally absorbed Pb via the portal system. Earlier studies on Pb-induced hepatotoxicity revealed alterations in hepatic biotransformation, metabolism of cholesterol, hepatocyte hyperproliferation, and nucleic acid synthesis due to hepatocyte hyperplasia caused by Pb [4–5]. Previous studies indicate that Pb-induced hepatotoxicity is mediated by free radicals [5–7].

Lead is reported to cause lipid peroxidation (LPO) in the liver and deplete glutathione (GSH). Consistently, Pb has been found to derail the antioxidant defense system by interfering with the metals that are essential for antioxidant enzyme activities, supporting the role of oxidative stress in lead hepatotoxicity [8]. In view of the oxidative stress induction by Pb, a therapeutic strategy to elevate the antioxidant defenses of the body may be of assistance in protecting from Pb toxicity.

*Indigofera oblongifolia* has been used to alleviate pain due to its analgesic and anti-inflammatory properties. *Indigofera oblongifolia* (*I*. *oblongifolia*; hasr in Arabic) belongs to the family Fabaceae and is widely distributed in Asia and Africa. The plant contains the novel molecules known as indigin and indigoferic acid as alkylated xanthenes and the fatty acid ester of p-hydroxy (E)-cinnamic acid, respectively. β-sitosterol and 3-hydroxybenzoic acid were also found in the plant [9]. *I*. *oblongifolia* protects hepatocytes from carbon tetrachloride-induced hepatotoxicity through the suppression of oxidative stress-induced membrane lipid, nuclear DNA, and protein oxidation [10].

To our knowledge, there are no similar studies available on the preventive effects of *Indigofera oblongifolia* leaf extract on Pb-induced hepatotoxicity in rats. In view of this, the present study was aimed to elucidate whether *Indigofera oblongifolia* leaf extract (IOLE), when pre-administered before lead acetate treatment, can prevent oxidative stress-induced hepatotoxicity.

## Materials and Methods

### Chemicals and experimental animals

Lead(II) acetate trihydrate (Pb(CH_3_CO_2_)_2_ · 3H_2_O; CAS Number 6080-56-4), nitro blue tetrazolium, N-(1–naphthyl) ethylenediamine, 5,5`-dithiobis (2-nitrobenzoic acid), nicotinamide adenine dinucleotide phosphate (NADPH), and Tris—HCl were purchased from Sigma (St. Louis, MO, USA). Thiobarbituric acid (TBA) and trichloroacetic acid were purchased from Merck. All other chemicals and reagents used in this study were of analytical grade. Double-distilled water was used as the solvent.

Eight week old male Wistar albino rats weighing 150–180 g were purchased from the animal facility of the Holding Company for Biological Products and Vaccines (Cairo, Egypt), and housed in wire polypropylene cages in a room under standard laboratory conditions (12 hrs light-dark cycle; 25±2°C). The rats were provided water and an animal standard diet *ad libitum* and allowed to acclimatize one week prior to the experiment. All of the experimental procedures were conducted according to the European Community Directive (86/609/EEC) the national rules on animal care that was carried out in accordance with the NIH Guidelines for the Care and Use of Laboratory Animals 8^th^ edition, and were approved by the Institutional Animal Ethics Committee guidelines for animal care and use at Helwan University.

### Plant extract

*I*. *oblongifolia* leaves were collected from the Jazan Province in the southwest region of the Kingdom of Saudi Arabia with the following geographical coordinates: 16°58′08″N 42°49′57″E. Collection was done in the month of October, 2014. The leaves were taken from *I*. *oblongifolia* trees growing abundantly in the open field of the local residents belonging to a small community after the proper consent was secured from the owners of the lands. The identity of this species was confirmed by Dr. Pandalayil (Botany department, College of Science, King Saud University). Leaves were rinsed under running tap water to remove dust, air dried at a temperature not exceeding 40°C, and ground into a powder using a pulverizer. The powdered leaves were extracted with water-methanol (1:2). In brief, the powder was incubated at 4°C for 24 h with mixing from time to time. IOLE was filtered through Whatman grade 1 filter paper to remove any debris. The filtrate was then evaporated to dryness in a vacuum evaporator (Heidolph, Germany). Residues were dissolved in water and were protected from light at -20°C until used in this study. The percentages of phenolic compounds and flavonoids were determined in the IOLE using standard methods.

### HPLC-ESI-MS analysis

Three replicates from the IOLE were centrifuged in an Eppendorf tube (2 min at 1400 rpm) and filtered through a 0.45-μm filter. A liquid chromatography apparatus 1290 series from Agilent Technologies, including a degasser, a binary pump delivery system, and an automatic liquid sampler, was used and coupled to Agilent Triple Quad Model 6460 mass spectrometer detectors. The HPLC column was a ZORBAX Eclipse plus C18 (4.6 × 150 mm, 5 μm) from Agilent Technologies (Agilent Technologies, Palo Alto, CA, USA). Elution was carried out using acetic acid (2%; A) and acetonitrile (B). The elution gradient used was as follows: 0 min, 5% B; 2 min, 7% B; 4 min, 9% B; 6 min, 12% B; 8 min, 15% B; 9 min, 16% B; 10 min, 17% B; 11 min, 17.5% B; 12 min, 18% B; 14 min, 20% B; 16 min, 28% B; 18 min, 100% B; 22 min, 100% B; 23 min, 5% B. The initial conditions were maintained for 5 min. The flow rate was set at 0.80 ml/min throughout the elution. Separation was carried out at 30°C. MS analysis was carried out using the electrospray ionization (ESI) interface in negative ionization mode, and mass detection was performed in the full scan mode in the range of *m/z* 50–2000 *m/z*. The following settings were applied to the instrument: capillary voltage, 4000 V; end plate offset −500 V.

### Experimental design

To study the protective effects of IOLE on lead acetate (PbAc)-induced hepatotoxicity, the rats were randomly divided into two groups: the control group (Control group; n = 7) and the experimental group (n = 21). The rats in the experimental group were divided into three groups: IOLE alone, PbAc alone, and IOLE with PbAc, with each group consisting of seven rats. The control group was administered 0.3 ml of saline orally, followed by intraperitoneal injection (i.p.) with 100 μL of saline after 1 h. IOLE was administered orally daily at 9:00 AM to non-fasted rats at a dose of 100 mg/kg bwt according to Lubbad et al. [11], while PbAc was injected i.p. daily at a dose of 20 mg/kg bwt according to Abdel Moneim [12] to induce acute toxicity in rats. The IOLE was pre-administrated 1 h before the PbAc injection.

The animals were sacrificed under mild ether anesthesia 24 h after last administration. Blood was collected from the abdominal aorta using a syringe puncture, and the serum was separated. The liver was carefully removed and washed twice in ice-cold 50 mM Tris—HCl, pH 7.4. Then, each liver was weighed and immediately homogenized in ice-cold medium containing 50 mM Tris—HCl (pH 7.4) to give a 10% (w/v) homogenate. The homogenates were centrifuged at 1000 × *g* for 10 minutes at 4°C. The supernatants were used for the various biochemical determinations. The total protein content of the homogenized liver was determined by the method of Lowry et al. [13] using bovine serum albumin as a standard.

### Lead concentration in the liver

Lead concentration was measured in the liver tissue using the method of Szkoda and Zmudzki [14]. Liver tissue samples were dried in an oven at 60°C and combusted at 450°C for 24 h. Thereafter, the combusted samples were dissolved in a hot solution of 1 M HNO_3_. The samples were transferred into 50 ml volumetric flasks and adjusted to 50 ml with deionized water. The appropriately diluted and digested tissue samples were analyzed at 283.3 nm using a flame atomic absorption spectrophotometer (Perkin-Elmer, 3100). The amount of lead in the liver was expressed as μg/g wet tissue weight.

### Changes in the liver index of rats

The weight of each liver was calculated as liver weight/body weight × 100.

### Liver function and lipid profile tests

The presence of transaminases [alanine aminotransferase (ALT) and aspartate aminotransferase (AST)] in the sera was estimated according to the method of Reitman and Frankel [15], by measuring the amount of pyruvate or oxaloacetate produced by reaction with 2,4-dinitrophenylhydrazine spectrophotometrically at 546 nm. The Belfield and Goldberg method was used to assay the levels of alkaline phosphatase (ALP), using kits provided by Randox Laboratories Co. [16]. The Schmidt and Eisenburg method was employed to determine the total bilirubin in the sera [17]. Serum triacylglycerols (TG), total cholesterol (TC), low density lipoprotein cholesterol (LDLc), and high density lipoprotein cholesterol (HDLc) were determined with commercially available diagnostic kits (Biodiagnostic-Egypt) as per the manufacturer’s instructions.

### Oxidative stress markers in the liver

Lipid peroxidation (LPO) in the hepatic homogenate was determined using 1 ml of 0.67% thiobarbituric acid and 1 ml of 10% trichloroacetic acid in a boiling water bath for 30 min. Thiobarbituric acid reactive substances were determined by absorbance at 535 nm and expressed as the amount of malondialdehyde (MDA) formed [18]. The level of hydrogen peroxide (H_2_O_2_) in the hepatic homogenate was determined using 3,5-dichloro-2-hydroxybenzensulfonic acid and 4-aminophenazone, with the resultant chromophore being measured at 405 nm. The level of nitric oxide in the liver was determined by the optimized acid reduction method in an acidic medium and in the presence of nitrite. This reaction couples nitrous acid diazotized sulfanilamide with N-(1–naphthyl) ethylenediamine, and the resultant bright reddish purple azo dye can be measured at 540 nm [19]. In addition, the level of hepatic glutathione (GSH) was determined by the reduction of 5,5`-dithiobis (2-nitrobenzoic acid) (Ellman's reagent) with GSH to form a yellow compound. The amount of reduced chromogen is directly proportional to the GSH concentration and its absorbance can be measured at 405 nm [20].

### Antioxidant status in the liver

The activity of hepatic superoxide dismutase (SOD) was assayed by measuring the ability of the enzyme to inhibit the phenazine methosulfate-mediated reduction of nitroblue tetrazolium (NBT) dye. Hepatic catalase (CAT) was assayed by adding 50 μl of liver homogenate to 30 mM H_2_O_2_ in 50 mM of potassium phosphate buffer (pH 8.0), and the decomposition of H_2_O_2_ was measured at 340 nm for 120 s at 20 s intervals. Catalase activity was expressed in units. Glutathione reductase (GR) activity was assayed indirectly by GR catalysis of the reduction of glutathione in the presence of NADPH, which is oxidized to NADP^+^. The decrease in absorbance at 340 nm was measured. Finally, the hepatic glutathione peroxidase (GPx) activity was measured using the method of Paglia and Valentine [21]. The assay is an indirect measure of the activity of GPx. Oxidized glutathione, produced upon reduction of organic peroxide by GPx, is recycled to its reduced state by the enzyme glutathione reductase. The oxidation of NADPH to NADP^+^ is accompanied by a decrease in absorbance at 340 nm.

### Quantitative real time PCR

Total RNA was purified from the liver tissue using an RNeasy Plus Minikit (Qiagen, Valencia, CA). cDNA was prepared using the RevertAid^™^ H Minus Reverse Transcriptase (Fermentas, Thermo Fisher Scientific Inc., Canada). The cDNA samples were run in triplicate for real-time PCR analysis. Real-time PCR reactions were performed using Power SYBR^®^ Green (Life Technologies, CA) on an Applied Biosystems 7500 system. Relative values of gene expression were normalized to β-actin. Primer sequences and accession numbers of the genes are provided in Table 1.

**Table 1:** Primer sequences of genes analyzed by real-time PCR.

Name	Accession number	Sense (5'---3')	Antisense (5'---3')
β-actin	NM_031144.3	GGCATCCTGACCCTGAAGTA	GGGGTGTTGAAGGTCTCAAA
SOD2	NM_001270850.1	AGCTGCACCACAGCAAGCAC	TCCACCACCCTTAGGGCTCA
CAT	NM_012520.2	TCCGGGATCTTTTTAACGCCATTG	TCGAGCACGGTAGGGACAGTTCAC
GPx	NM_017006.2	CGGTTTCCCGTGCAATCAGT	ACACCGGGGACCAAATGATG
Nrf2	NM_031789.2	GGTTGCCCACATTCCCAAAC	GGCTGGGAATATCCAGGGC
HO-1	NM_012580.2	GCGAAACAAGCAGAACCCA	GCTCAGGATGAGTACCTCCCA
Bcl-2	NM_016993.1	CTGGTGGACAACATCGCTCTG	GGTCTGCTGACCTCACTTGTG
Bax	NM_017059.2	GGCGAATTGGCGATGAACTG	ATGGTTCTGATCAGCTCGGG

SOD2: Manganese-dependent superoxide dismutase (MnSOD); CAT: Catalase; GPx: Glutathione peroxidase; Nrf2: Nuclear factor erythroid 2–related factor 2; HO-1: Heme oxygenase 1; Bcl-2: B-cell lymphoma 2; Bax: Bcl-2-like protein 4.

### Histological changes

The liver was fixed in 10% neutral buffered formalin for 24 h, dehydrated in ethyl alcohol, cleared in xylene, and mounted in molten paraplast. Sections of 4–5 μm were obtained from the prepared blocks and stained with hematoxylin-eosin. Sections were examined using a Nikon (Eclipse E200-LED, Tokyo, Japan) microscope.

### Apoptotic and fibrotic markers in the liver tissue

Liver tissue samples were fixed in neutral buffered formalin (10%), embedded in paraffin, and sectioned into 4 μm sections. Immunocytochemical reactions were performed using the peroxidase/anti-peroxidase (PAP) method [22]. Non-specific peroxidase reactions were blocked with methanol containing 0.1% H_2_O_2_. The sections were also incubated with normal goat serum to avoid non-specific reactions once the samples were incubated with specific antibodies against Bcl-2, Bax, caspase-3 or matrix metalloproteinase-9 (dilution, 1:2000, Santa Cruz CA, USA). Tissue sections were then washed with phosphate buffer and incubated with secondary antibodies (1:2000; Sigma, USA), before being washed in phosphate buffer again and, finally, incubated with the PAP complex (dilution, 1:200). The peroxidase reaction was carried out using a solution of 3,3'-diaminobenzidine tetrahydrochloride containing 0.01% H_2_O_2_ in Tris—HCl buffer (0.05 M, pH 7.6). After immunostaining, the liver sections were lightly counterstained with hematoxylin and observed under a light microscope.

### Statistical analysis

All results were expressed as the mean ± standard error of the mean (SEM). Data for multiple variable comparisons were analyzed by one-way analysis of variance (ANOVA). For the comparison of significance between groups, Duncan's test was used as a post hoc test, and probability level of p<0.05 was considered significant.

## Results

**Fig 1:**
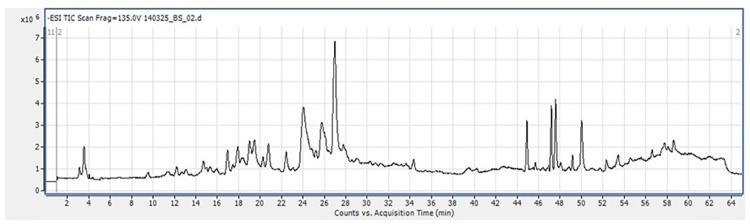
HPLC-ESI-MS chromatogram of *Indigofera oblongifolia* leaf extract.

In the present study, 27 different compounds were detected in the IOLE using electrospray ionization mass spectrometry (ESI-MS) as shown in Fig 1. A broad range of polyphenols, flavonoids, and organic acids were detected in the extract. The retention times and molecular ion [M-H]^−^ along with fragment ion peaks are described in S1 Table. The IOLE contains **anthocyanidins** (cyanidin 3-O-[2"-O-(2‴-O-(sinapoyl)xylosyl)glc]5-O-glc and cyanidin 3-O-[2"-O-xylosyl-6"-O-(p-coumaroyl)glucoside]5-O-malonylglc), **glucosinolates** (1-methoxy indolyl glutathione, 5-benzoyloxypentyl glucosinolate, and indol-3-ylmethyl glucosinolate), **flavones** (apigenin 6-C-glucoside, luteolin 3,7′-di-O-glucoside, lupinisoflavone, apigenin-7-O-glucoside, luteolin C-glucoside C-xyloside, luteolin, and luteolin C-6-(2" O-rhamnosyl)glucoside), **polyamines** (caffeoyl putrescine and diferuloyl spermine), **flavonols** (quercetin mono-sinapoyl-di-O-[glc or gal], quercetin- rhamnoside dimer 1, kaempferol 3-O-[rhamnosyl-glucosylglucoside] 7-O-rhamnoside, 3'-O-methylluteolin 6-C-glucoside, and methyl-O-quercetin rhamnosylglucoside), **phytosterols** (β-sitosterol glucoside and acylated (16:0) β-sitosterol glucoside), **vanillic acid**, and **hydroxycinnamic acid ester** (1,2,2'-trisinapoyl gentiobiose). In addition, the IOLE contains alkaloids such as indigotin, indigo, and an isomer of indirubin. Furthermore, the yield of total polyphenols from the IOLE was 18.2% and the yield of flavonoids was 8.4% (S1 Fig).

**Fig 2:**
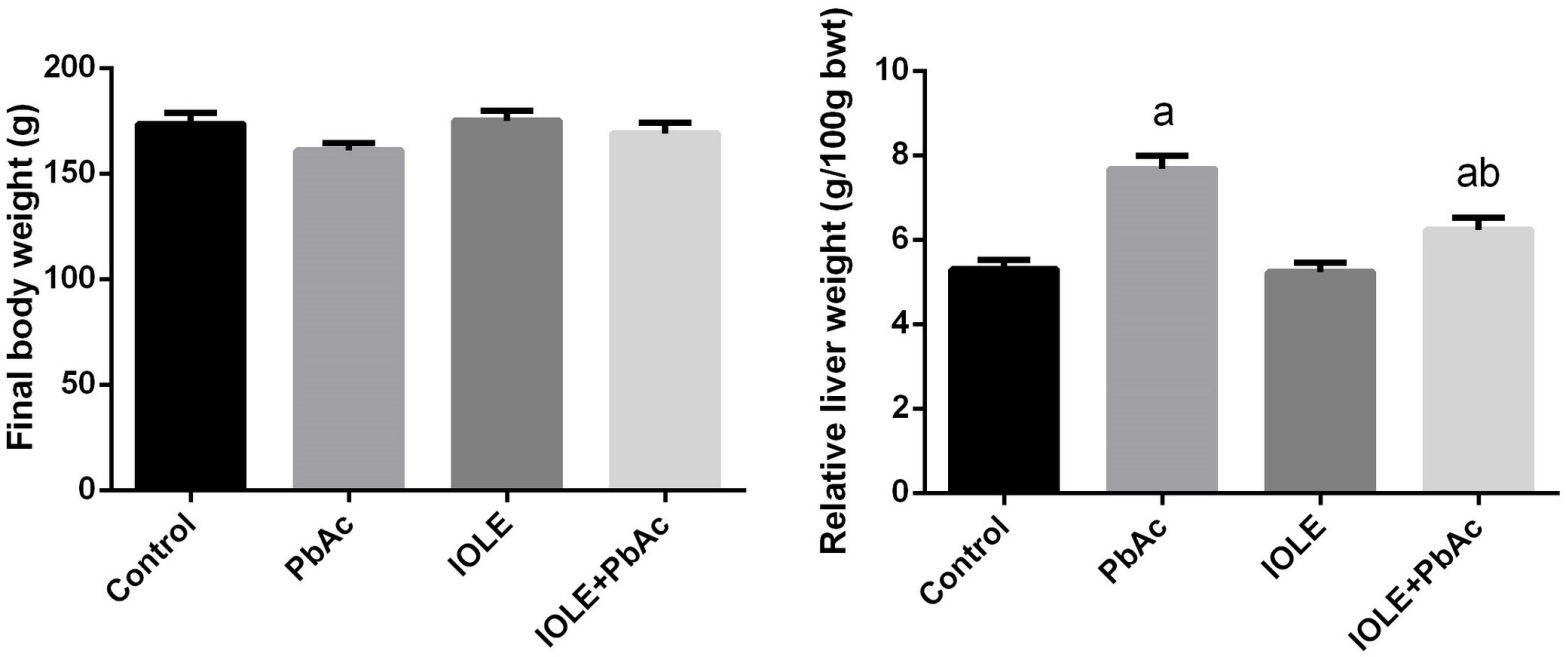
Preventive effects of *I*. *oblongifolia* leaf extract (IOLE) on the final body weight and relative liver weight of rats treated with lead acetate (PbAc) for 5 days. All results are expressed as the mean ± SEM (n = 7). ^a^*p*<0.05, significant change with respect to Control; ^*b*^*p*<0.05, significant change with respect to PbAc using Duncan's post hoc test.

The data illustrated in Fig 2 showed that rats injected with PbAc alone had no significant (p<0.05) change in the final body weight, while a significant (p<0.05) elevation in liver weight was observed compared with the control rats. Conversely, rats pre-treated with the IOLE exhibited an improvement in the relative liver weight but this change was significantly increased compared with the control rats.

**Fig 3:**
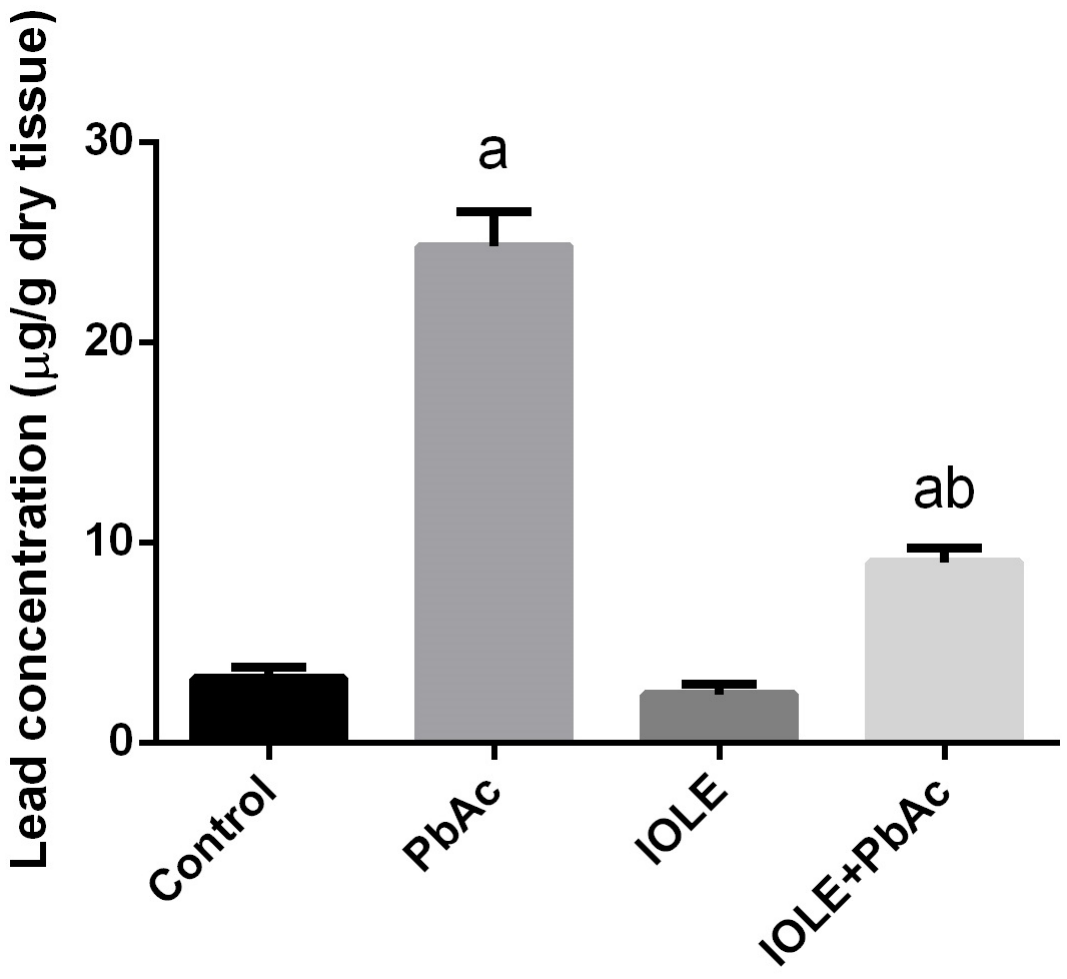
Protective effects of *I*. *oblongifolia* leaf extract (IOLE) on lead accumulation in liver tissue of rats treated with lead acetate (PbAc) for 5 days. All results are expressed as the mean ± SEM (n = 7). ^a^*p*<0.05, significant change with respect to Control; ^*b*^*p*<0.05, significant change with respect to PbAc using Duncan's post hoc test.

In rats treated with PbAc for 5 days, the Pb^2+^ content of the liver was significantly (p<0.05) increased in comparison with the control group (Fig 3). However, when the rats were pre-administered with the IOLE 1 h before PbAc, the Pb^2+^ content of the liver was significantly lower in comparison with the rats treated with PbAc.

**Fig 4:**
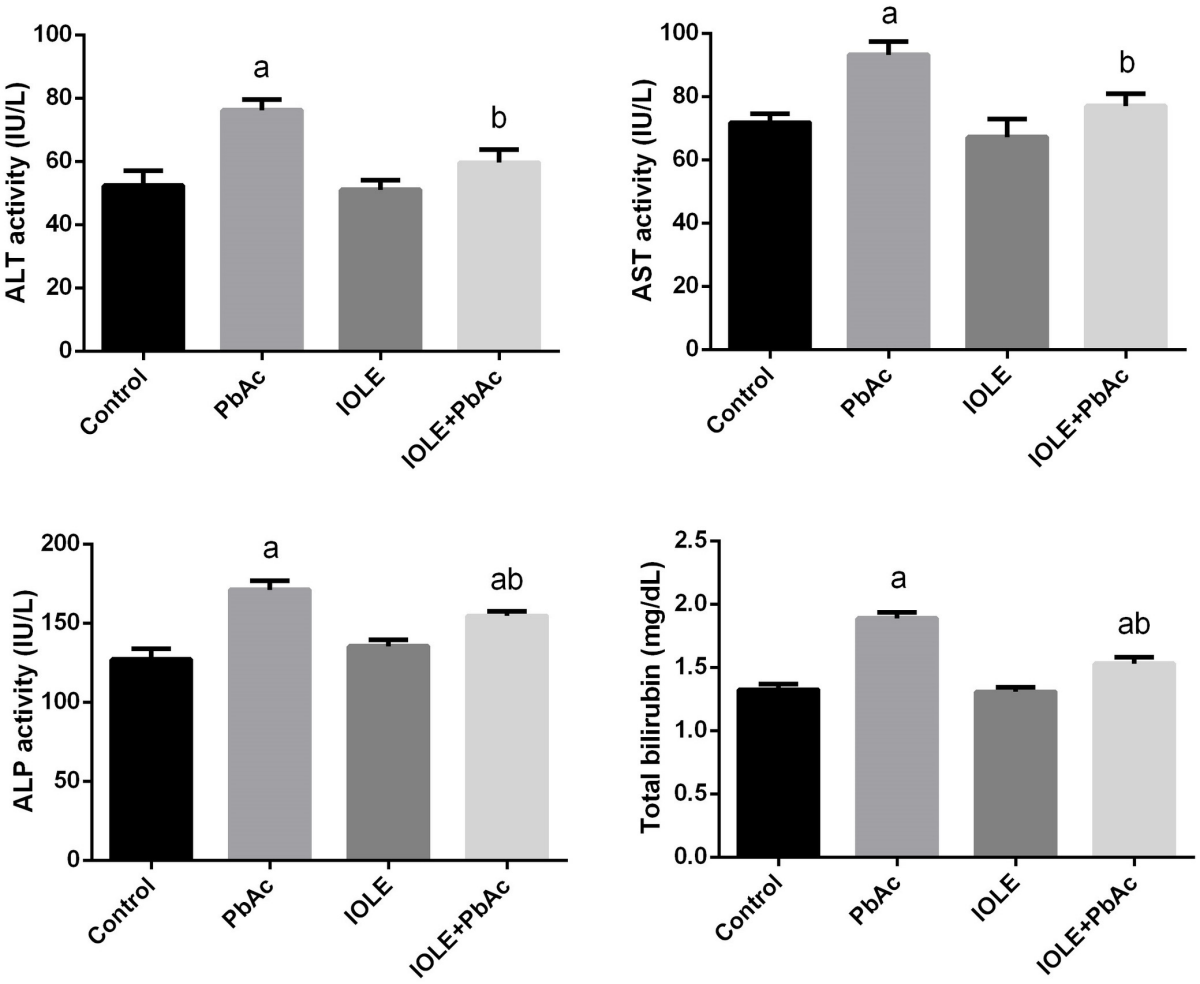
Ameliorative effects of the pre-administration of *I*. *oblongifolia* leaf extract (IOLE) on alanine aminotransferase (ALT), aspartate aminotransferase (AST), alkaline phosphatase (ALP), and total bilirubin in rats exposed to lead acetate (PbAc) for 5 days. All results are expressed as the mean ± SEM (n = 7). ^a^*p*<0.05, significant change with respect to Control; ^*b*^*p*<0.05, significant change with respect to PbAc using Duncan's post hoc test.

Following PbAc treatment of rats, the activity of serum transaminases (ALT and AST), ALP, and total bilirubin was significantly (p<0.05) increased in comparison with the control rats (Fig 4). Pre-administration of rats with the IOLE prevented the liver function parameters from rising, but ALP activity and total bilirubin levels were significantly increased compared with the control rats.

The PbAc-induced hepatotoxicity resulted in a significant elevation (p<0.05) in serum triglyceride, cholesterol, LDLc, and reduction in HDLc levels compared with the control rats as shown in Table 2. However, when the PbAc rats were pre-treated with the IOLE, a significant recovery was evident, compared with the control, with the exception of serum triglyceride and HDLc levels.

**Table 2:** Serum lipid profile in rats treated with IOLE and/or PbAc for 5 days.

Experimental groups	Triglyceride (mg/dL)	Cholesterol (mg/dL)	LDLc (mg/dL)	HDLc (mg/dL)
**Control**	50.7±5.51	140.3±10.41	91.5±7.16	42.3±3.72
**PbAc**	70.3±6.27^a^	169.7±12.34^a^	113.8±9.23^a^	28.3±2.17^a^
**IOLE**	52.3±3.22	149.7±8.74	85.3±5.18	46.2±5.47
**IOLE-PbAc**	58.9±4.36^a^^b^	151.3±13.63^b^	98.3±8.61^b^	34.6±2.65^a^^b^

Values are mean ± SEM (n = 7)

^a^*p*<0.05, significant change with respect to Control group;

^b^*p*<0.05, significant change with respect to PbAc group using Duncan's post hoc test.

**Fig 5:**
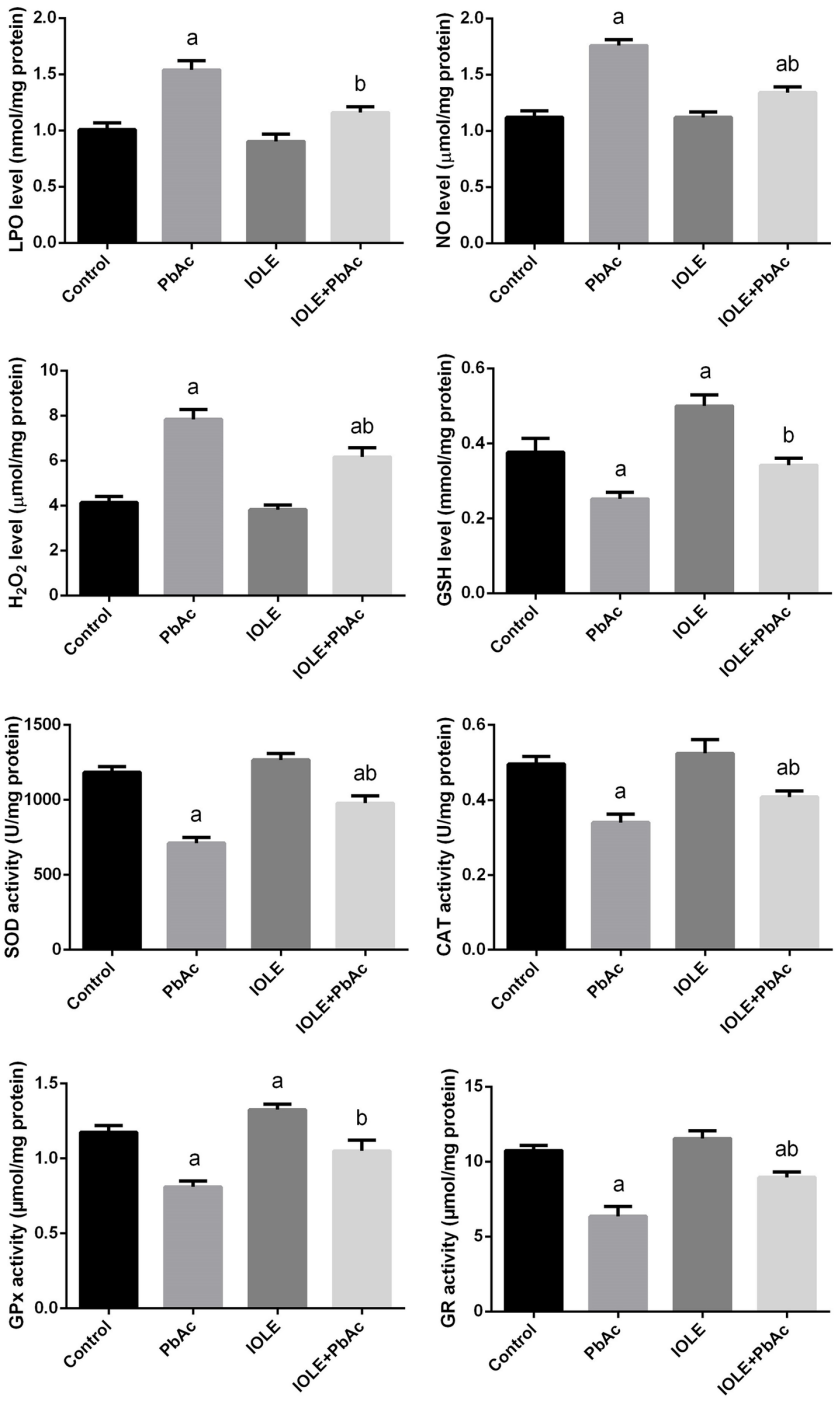
Ameliorative effects of the pre-administration of *I*. *oblongifolia* leaf extract (IOLE) on the oxidative stress markers levels and antioxidant enzyme activities in rats exposed to lead acetate (PbAc) for 5 days. All results are expressed as the mean ± SEM (n = 7). ^a^*p*<0.05, significant change with respect to Control; ^*b*^*p*<0.05, significant change with respect to PbAc using Duncan's post hoc test.

**Fig 6:**
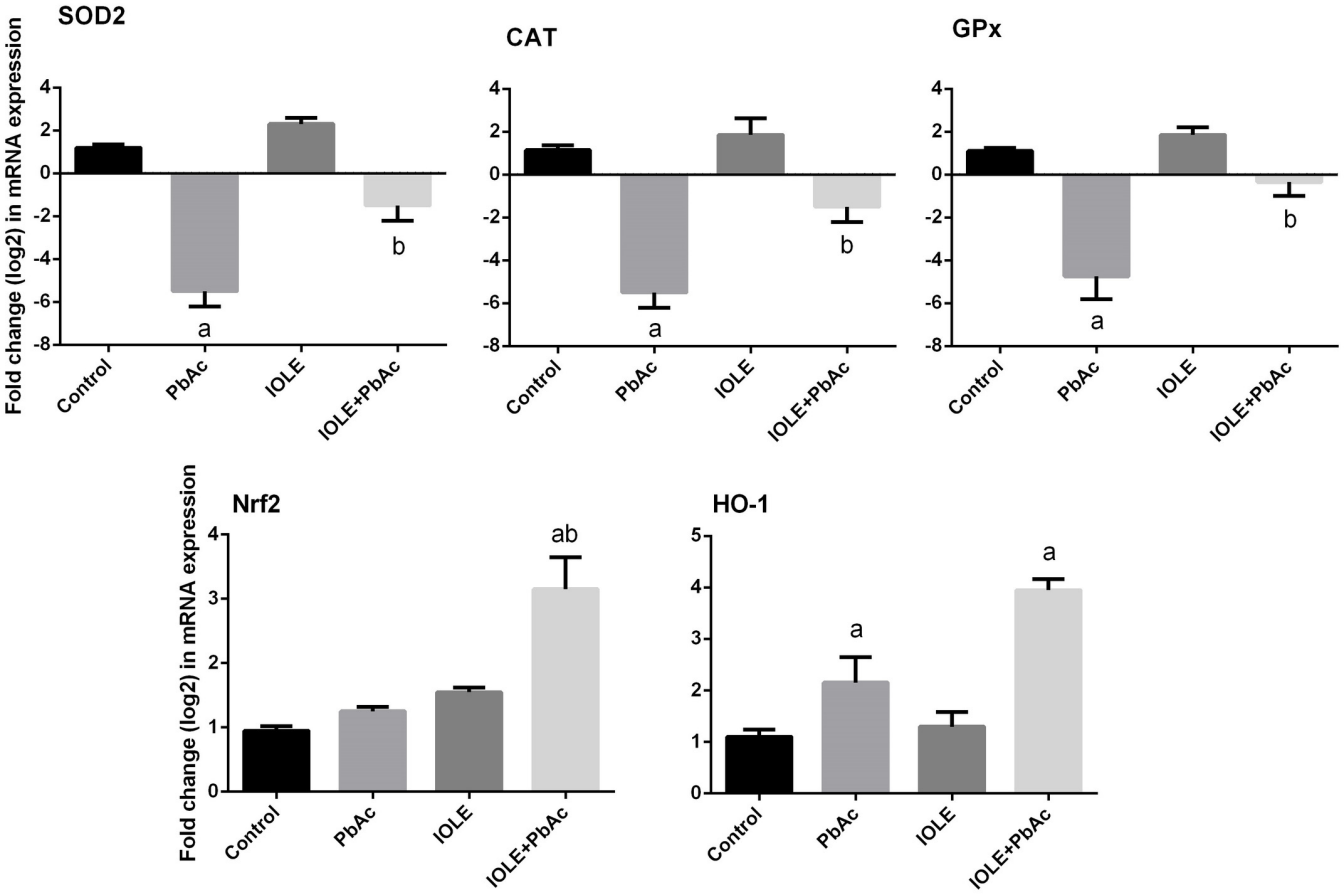
Ameliorative effects of the pre-administration of *I*. *oblongifolia* leaf extract (IOLE) on the mRNA levels of SOD2, CAT, GPx,Nrf2 and HO-1 genes in the liver of rats exposed to lead acetate (PbAc) for 5 days. All results are expressed as the mean ± SEM (n = 7). ^a^*p*<0.05, significant change with respect to Control; ^*b*^*p*<0.05, significant change with respect to PbAc using Duncan's post hoc test.

The present study revealed that PbAc-induced oxidative stress in the liver as indicated by a significant elevation in hepatic oxidative stress markers, including MDA and NO in the liver (Fig 5; p<0.05). Meanwhile, obvious declines in enzymatic (SOD, CAT, GPx, and GR) and non-enzymatic (GSH) antioxidant molecules were found when compared with the control group (Fig 5; p<0.05). In contrast, the pre-treatment of rats with the IOLE significantly attenuated the reduction of antioxidant molecules and the formation of MDA and NO in the liver. Consistent with the biochemical results, the quantitative real-time PCR data showed that the mRNA levels of SOD2, CAT, and GPx were down-regulated after PbAc injection; while, the IOLE was able to up-regulate these genes (Fig 6). The present study also determined the effect of IOLE on the Nrf2-antioxidant system in the liver of rats. PbAc did not promote Nrf2 transcription in the liver. However, Nrf2 mRNA level was promoted in the IOLE+PbAc group. Furthermore, qRT-PCR data showed that HO-1 mRNA level was up-regulated after PbAc treatment. Moreover, IOLE pre-administration further promoted HO-1 expression compared with that in PbAc-treated rats (Fig 6).

**Fig 7:**
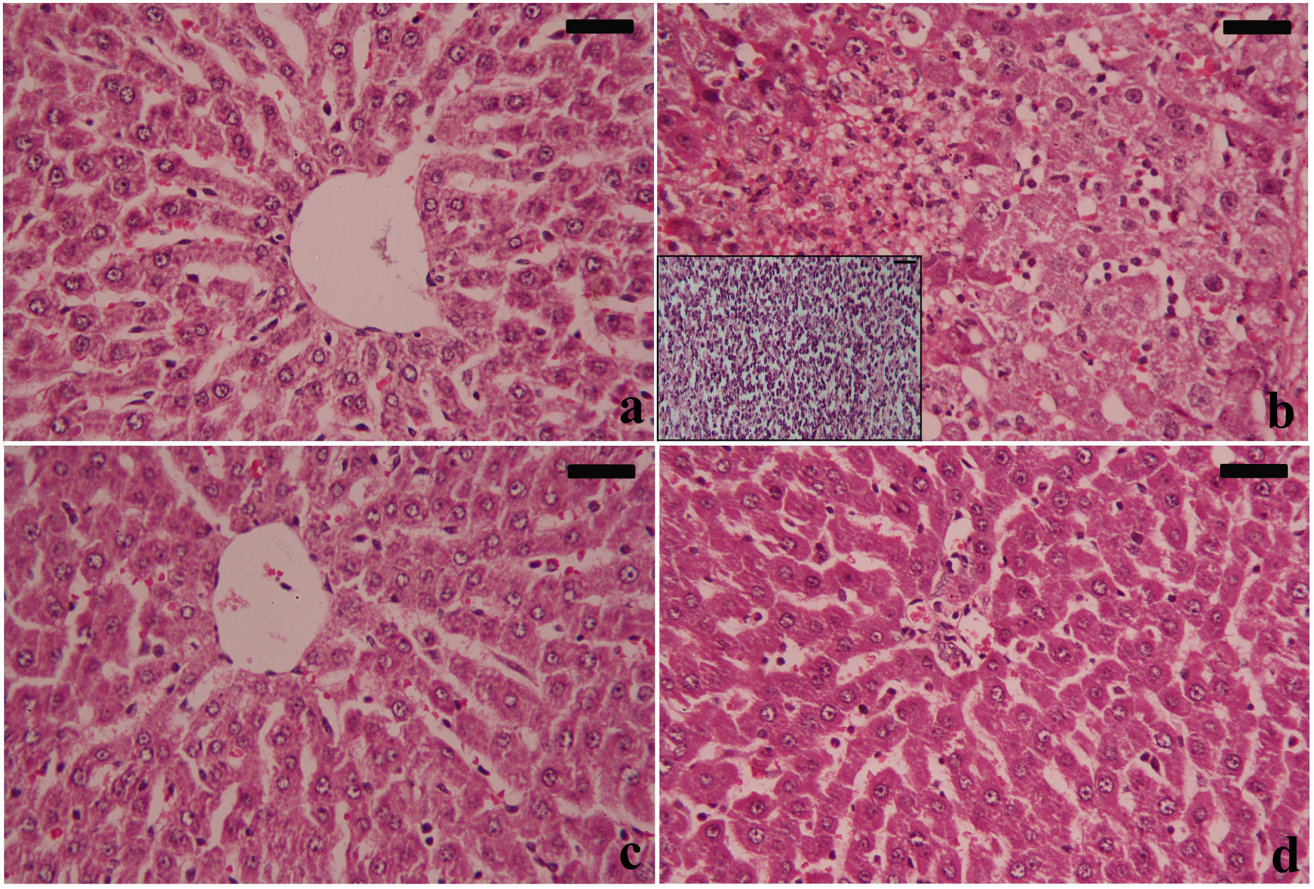
Light micrographs of the liver. a shows the normal histological structure of liver in the control group. b shows focal hepatic necrosis accompanied by dilated blood sinusoids and congested central vein, acute inflammatory cells infiltrating mainly in the central zone and extensive degeneration, cytoplasmic vacuolation, and steatosis in the PbAc-treated group, the inset in (b) shows focal neutrophil infiltrate associated with hepatocyte necrosis. c shows the normal histological structure of hepatocytes in the IOLE group. d shows the normal histological structure of hepatocytes in the PbAc-treated group pre-administered with IOLE. However, some vacuolated areas are seen (H & E). (scale bar 50 μm).

Normal histological architecture was observed in the liver sections of the control rats (Fig 7a). However, the liver sections of the PbAc group showed focal hepatic necrosis accompanied by dilated blood sinusoids and congested central veins. Infiltration of acute inflammatory cells was observed mainly in the central zone. Derangement of hepatocyte cords with pyknotic and karyolitic nuclei was found. Further, hepatocytes showed vacuolization and fatty change (steatosis), which included the intracellular accumulation of fats (Fig 7b). Treatment of rats with the IOLE largely improved the lead-induced histopathological changes in the hepatic tissue, showing reduction in the infiltration of inflammatory cells, vascular degeneration, and necrosis with fewer fat vacuoles (Fig 7d).

**Fig 8:**
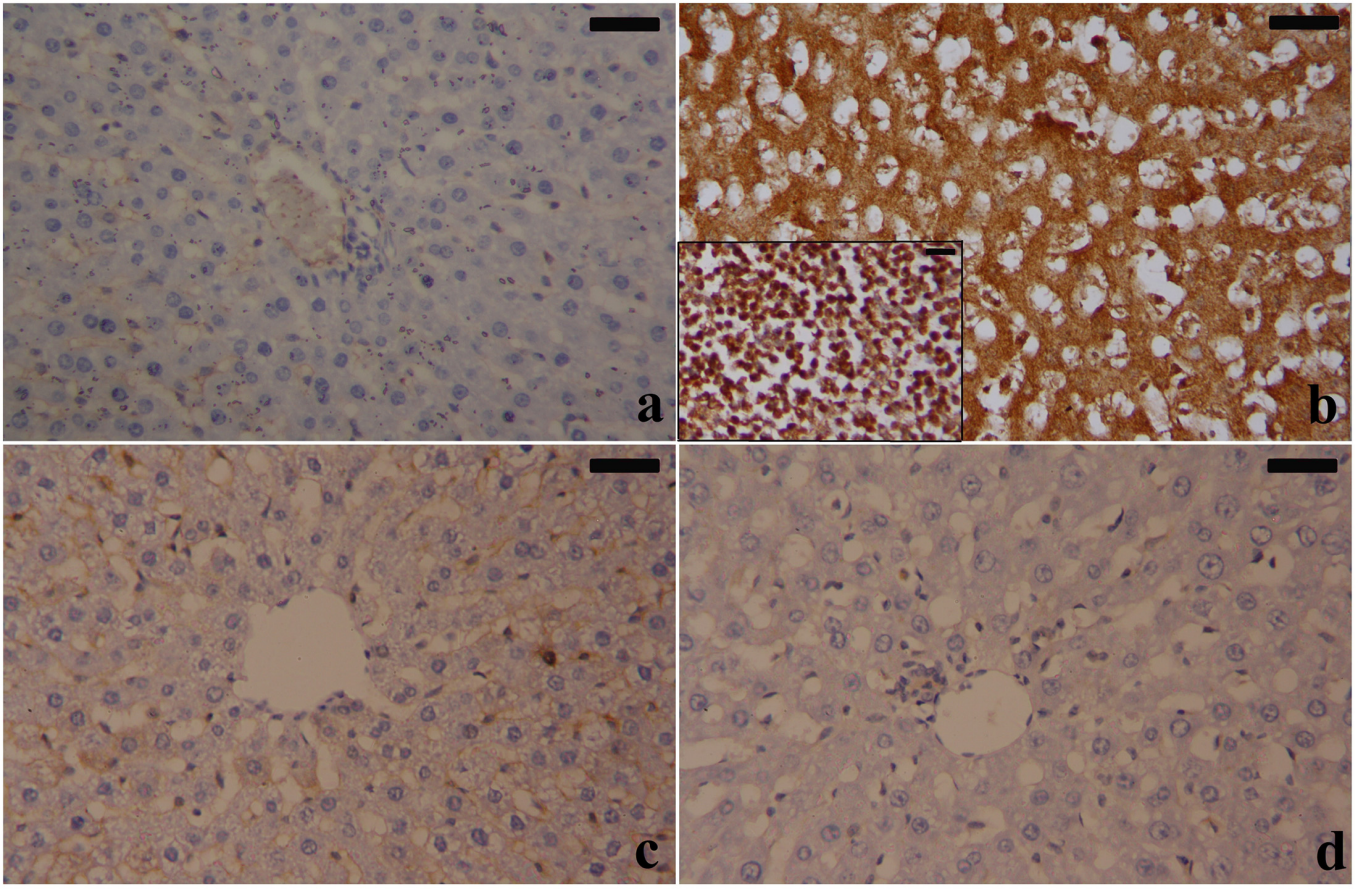
Changes in MMP-9 expression in rat hepatocytes after administration of IOLE and PbAc for 5 days. a-d show the morphology of hepatocytes in the control, PbAc, IOLE, and IOLE+PbAc treated rats, respectively (scale bar 50 μm). The inset in (b) shows focal neutrophil infiltration highly expressed MMP-9.

Matrix metalloproteinase-9 was found in the damaged areas of active fibrogenesis. Therefore, the present study examined the effect of IOLE on the expression of MMP-9. The immunohistochemical examination of control and IOLE livers showed weak immunoreactivity for MMP-9 in the hepatocytes (Fig 8a and 8c). In the PbAc-treated group, very strong MMP-9 immunoreactivity was observed in the hepatic tissues and especially in the inflammatory infiltrated cells (Fig 8b). IOLE treatments induced mild to moderate MMP-9 immunoreactivity in the hepatic tissues, indicating that the IOLE has anti-fibrous activity (Fig 8d).

**Fig 9:**
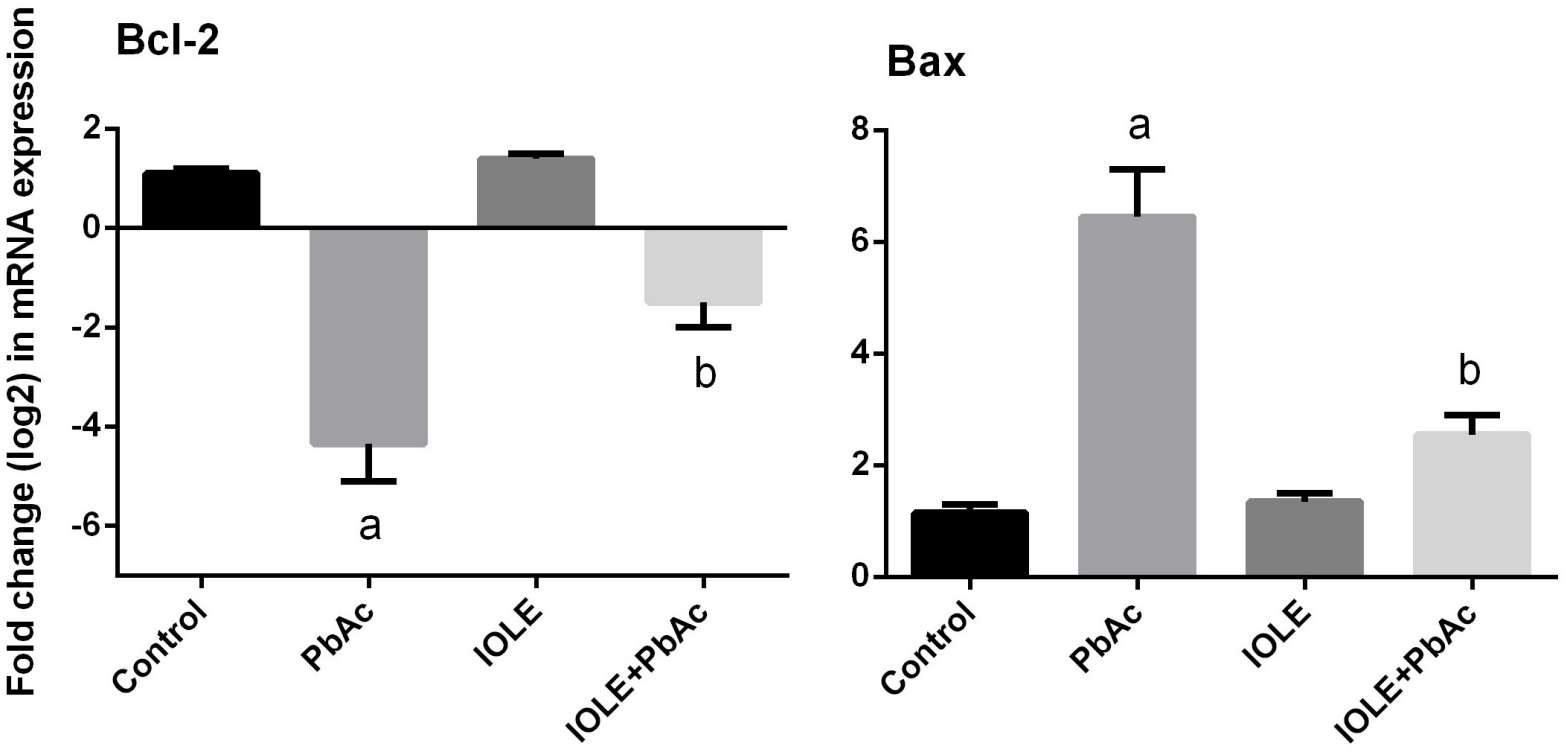
Ameliorative effects of the pre-administration of *I*. *oblongifolia* leaf extract (IOLE) on the mRNA levels of Bcl-2 and Bax in the liver of rats exposed to lead acetate (PbAc) for 5 days. All results are expressed as the mean ± SEM (n = 7). ^a^*p*<0.05, significant change with respect to Control; ^*b*^*p*<0.05, significant change with respect to PbAc using Duncan's post hoc test.

To investigate whether the observed hepatoprotective effects of IOLE were related to the antiapoptotic property of IOLE, the expression levels of *Bcl-2* and *Bax* in the liver were determined. The results revealed that the expression of *Bcl-2* mRNA was down-regulated markedly (Fig 9), whereas the expression of *Bax* was up-regulated in the PbAc treated rats (p<0.05). However, rats treated with IOLE prior to PbAc showed significant up-regulation of *Bcl-2* mRNA and down-regulation of *Bax*.

**Fig 10:**
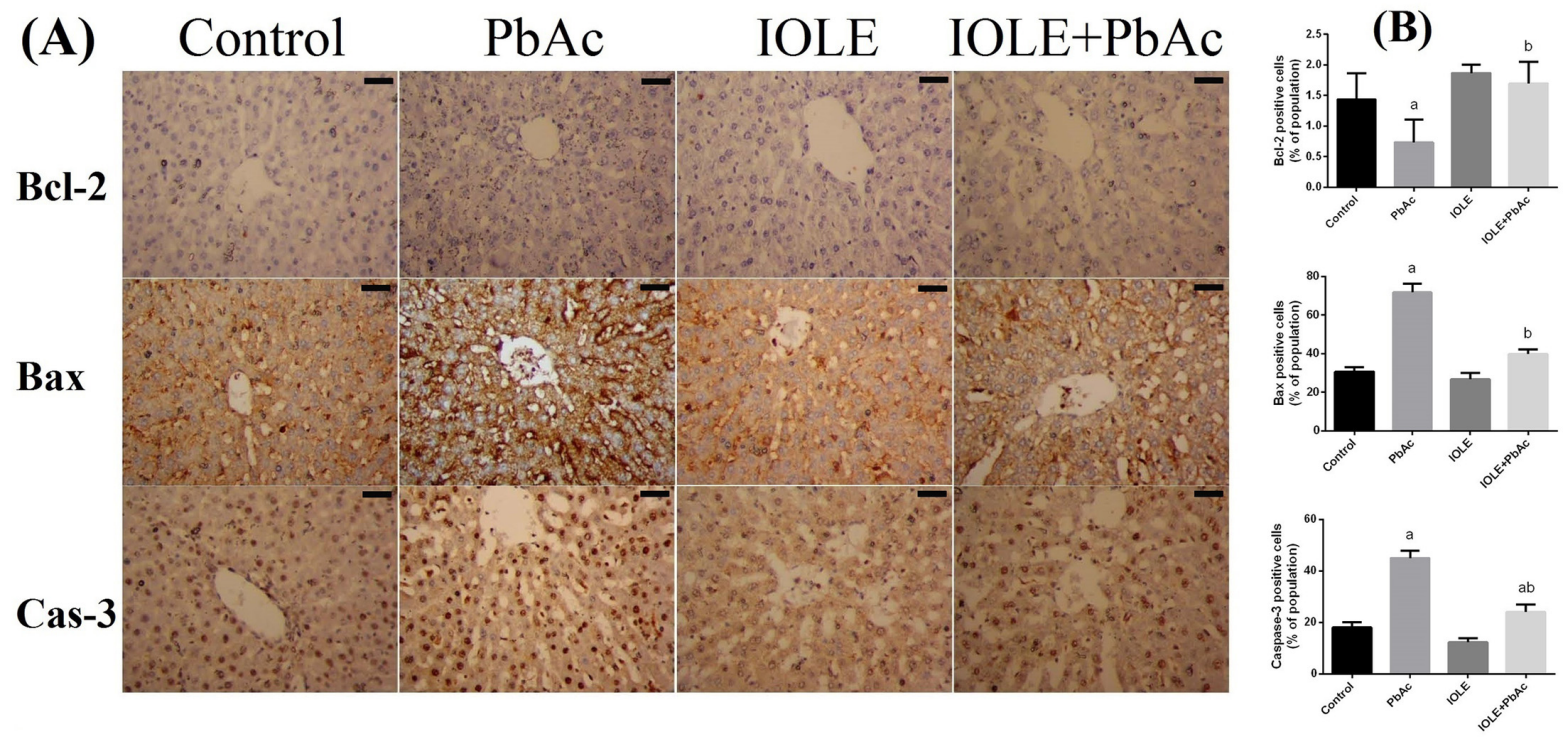
Bcl-2, Bax, and caspase-3 immunostained sections and morphometric analysis of hepatocytes in the control, PbAc-, IOLE-, and IOLE+PbAc-treated rats. (A) In the control and IOLE groups, apoptotic hepatocytes, i.e., those stained with Bax and caspase-3, were sparse and weakly stained. In the PbAc group, many hepatocytes were apoptotic. In the IOLE+PbAc group, the number of apoptotic hepatocytes was decreased. (scale bar 50 μm) (B) The plots show an increase in apoptotic hepatocytes in the control, PbAc-, IOLE-, and IOLE+PbAc-treated rats. All results are expressed as the mean ± SEM. A minimum of 20 high-magnification fields (× 400) per section were quantified. ^a^*p*<0.05, significant change with respect to Control; ^*b*^*p*<0.05, significant change with respect to PbAc using Duncan's post hoc test.

Consistent with real-time PCR, the immunohistochemical findings showed low immunoreactivity of Bcl-2 in the hepatocytes in PbAc-treated rats, while moderate to strong immunoreactivity was observed for Bax and caspase-3 (Fig 10). The pre-administration of IOLE to rats exhibited improvement, represented by increased expression of Bcl-2 protein in the hepatocytes and moderate expression of the pro-apoptotic proteins Bax and caspase-3 in the liver of rats treated with IOLE compared with the PbAc-treated rats.

## Discussion

No mortality was recorded in rats of control and experimental groups. No clinical or behavioural symptoms were noticed in the PbAc-treated rats. However, liver surface semblance was drastically altered, and the macromorphological changes included the loss of smooth and shiny color in the liver indicates liver fibrosis. In the current study, treatment with PbAc increased both absolute and relative liver weights radically, but did not affect the body weight significantly. The observed increase in the liver was thought to be due to necrosis and apoptosis, and could be attributed to the accumulation of the lipids and fibrous tissue in the liver. Treatment with IOLE, however, significantly mitigated these changes. These pathological features are typically observed in Pb intoxication because the liver, via the portal vein, is the first organ exposed to internally absorbed Pb and is one of the major organs implicated in the storage, biotransformation, and elimination of Pb.

The present findings revealed that Pb accumulates in the liver tissue, which was significantly ameliorated by pre-treatment with the IOLE. This indicates that the IOLE removes Pb from the liver by either chelating it and/or increasing its biotransformation. IOLE was also successful in preventing Pb accumulation in both testes [23] and kidney [24]. The IOLE is rich in flavonoids that act as metal chelators due to the presence of multiple hydroxyl groups forming a coordinate bond with Pb^2+^ [25]. However, the mechanism underlying the metal chelation property of the IOLE is not clear and needs further investigation.

The present data also revealed that treatment with PbAc caused a significant increase in ALT, AST, and ALP in serum, confirming the histological damage of the liver tissue. In fact, these enzymes are known as important markers of hepatocellular damage as affirmed by Abdel Moneim et al. [26]. These results are in accordance with the results of Abdou and Hassan [27]. Furthermore, the results also showed that PbAc significantly increased the serum levels of TC and TG in the PbAc group compared with the other groups. The increased levels of lipids along with increased levels of liver function markers may indicate liver dysfunction [28]. However, IOLE treatment resulted in a marked improvement of liver function markers and lipid profile parameters tested in the serum. The results indicated that the IOLE may stabilize the cellular membrane of hepatocytes and maintain its functions. In general, it appears that the potent hepatoprotector activity of IOLE may be attributed to its high phytochemical content.

An early event following liver injury by PbAc administration is the production of excessive amounts of reactive oxygen species (ROS), which induce hepatic stress by two separate, although related, pathways [29]. The first pathway is the ability of Pb to enhance lipid peroxidation, and the second pathway through the depletion of antioxidant reserves. Pb interacts with the sulfhydryl groups or metal cofactors in different enzymatic and non-enzymatic antioxidant molecules that results in a reduction in the activities of antioxidant enzymes and glutathione [30].

In the present work, PbAc impaired the antioxidant system, as the content of GSH, the main endogenous antioxidant, was depleted in association with increased lipid peroxidation, a marker of cellular oxidative stress that has long been recognized as a major consequence of oxidative damage in different diseases. MDA is the end product of lipid peroxidation. In the present study, elevated MDA levels were observed in the livers of PbAc-treated rats. Lipid peroxidation has traditionally been considered the major effect of free radicals, and lipid peroxidation products impair the physicochemical properties, fluidity, and integrity of cell membranes, thereby increasing susceptibility to lipid peroxidation and cell necrosis [31]. The IOLE reduces Pb-induced hepatotoxicity through an appreciable downregulation of lipid peroxidation.

In addition to oxidative stress, PbAc-induced hepatotoxicity may induce inflammation by activating Kupffer cells that play a major role in triggering a cascade of inflammatory mediators [32]. Furthermore, ROS can also promote inflammation by stimulating the transcription factor, nuclear factor kappa B (NF-κB), which in turn activates inducible NO synthase (iNOS) in macrophages [33]; this may explain the elevated levels of NO observed in the liver homogenates. The toxicity of NO increases greatly when it reacts with the superoxide radical, forming the highly reactive peroxynitrite anion (ONOO^−^). NO production is also positively associated with tissue fibrosis due to its induction of fibrogenic cytokines and increased collagen synthesis [34]. However, IOLE administration prevented the activation of NF-κB and the subsequent accumulation of NO. In fact, flavonoids found in IOLE have the ability to inhibit NF-κB transcription by blocking the phosphorylation and degradation of IκBα (nuclear factor of kappa light polypeptide gene enhancer in B-cells inhibitor, alpha) to decrease NF-κB DNA binding activity.

The protective effects of IOLE in maintaining GSH at near control levels should enhance the capacity of the endogenous antioxidant defense system. The IOLE may function by increasing the steady state levels of GSH and/or its rate of synthesis, thereby conferring enhanced protection against oxidative stress. GSH is a major non-enzymatic antioxidant, found both intracellularly and extracellularly in living organisms, which acts against xenobiotics and neutralizes ROS. In the present experiment, GSH content was decreased in PbAc-treated rats. This depletion in GSH content may contribute to the enhanced lipid peroxidation. Furthermore, Pb inactivates GSH synthesis from cysteine via the γ-glutamyl cycle, which further depresses the GSH content [29].

Reduced activities of antioxidant enzymes are frequently implicated in oxidative stress. Rats exposed to PbAc were reported to exhibit significantly lower levels of hepatic antioxidant enzymes. The present findings on antioxidant enzyme activities are in accordance with several previous studies that found significant reductions in antioxidant enzymes in the livers of rats exposed to Pb; these changes can be attributed to the numerous deleterious effects caused by the accumulation of superoxide radicals and hydrogen peroxide. Further, it has been documented that the Pb^2+^ ion competes with metal ions (such as Cu^2+^, Zn^2+^, Fe^2+^, and Mg^2+^) that are essential for the activity of antioxidant enzyme, resulting in a loss or decrease in antioxidant enzymes [35]. However, pre-treatment with IOLE prevented these enzymes from being downregulated, which suggests a regulatory role of IOLE on the antioxidant enzymes.

Nuclear factor erythroid 2–related factor 2 (Nrf2) was considered as the redox master regulator, and its promotion was in favor of protecting against oxidative stress. In the present study, the mRNA of Nrf2 was unchanged upon PbAc treatment which would in turn be expected to lower the expression of antioxidative enzymes, as found in the present study. Similarly, Ye et al. recently found that Nrf2 mRNA expression did not affect Nrf2 transcription in the hippocampus and frontal cortex [36]. However, Wang et al. demonstrated that exposure to Pb induced Nrf2 expression in testis [37]. IOLE pre-treatment increased the mRNA level of Nrf2. The ability of IOLE to induce Nrf2 may maintain sufficient transcriptional activation of various antioxidant genes to balance the redox homeostasis in liver, thereby promoting hepatocytes survival under Pb-exposed conditions. The present results confirmed the mechanisms of IOLE hepatoprotective effects. Additionally, the data mentioned that IOLE induced HO-1 in the liver.

Heme oxygenase 1 (HO-1) is an enzyme with important physiologically relevant antioxidant and cytoprotective activities and is commonly up-regulated following oxidative stress and cellular injury, and Nrf2 directly regulates its expression [38]. In the present study, HO-1 expression was up-regulated in the liver of rats exposed to PbAc. The increase in HO-1 expression may, in part, be an adaptive response that protects hepatocytes from PbAc-induced oxidative stress, suggesting that hepatocytes have the ability to restrain oxidative stress by HO-1 induction. The obtained data are in accordance with Yu et al. who found that PC12 cells protected themselves from lead acetate by promoting HO-1 expression [39]. IOLE pre-administration to PbAc-exposed rats further promotes the HO-1 expression. The present results demonstrated that promotion of HO-1 expression is required for IOLE antioxidative effects, but restrained HO-1 expression is not required for PbAc evoked oxidative stress in liver.

In this study, the expression levels of *caspase-3* and *Bax* were significantly upregulated in the liver at both mRNA and protein levels, while the levels of Bcl-2 mRNA and protein were downregulated significantly. Yuan et al. [40] reported that hepatic apoptosis induced by low-dose exposure to lead was associated with mitochondrial injury and changes in levels of apoptogenic proteins including *Bcl-2*, *Bax*, and *caspase-3*. Further, Flora and his colleagues demonstrated that increased ROS levels may cause an imbalance in Ca^2+^ regulation, which in turn induces an imbalance in anti- and proapoptotic proteins, such as *Bcl-2* and *Bax*, leading to the release of cytochrome *c* from mitochondria for the activation of the terminal cascades of apoptosis [41]. Conversely, the IOLE was significantly effective in reversing apoptosis in the liver. The protective effect of IOLE on the liver tissue is related to the inhibition of apoptosis, based on the ability of different phytochemicals to protect against stress-induced apoptosis.

The present study demonstrated that the PbAc treated rats showed an upregulation in the MMP-9 protein in the liver tissue. These results are in accordance with those of Lahat et al. [42], who found that exposure to lead activated the expression of MMP-9 in glial cells. Matrix metalloproteinases (MMPs) are secreted extracellular endopeptidases that can modify extracellular matrix (ECM) components and control cell behavior. MMP-9 is considered a hallmark of fibrosis, and its expression up-regulated by tumor necrosis factor alpha (TNF-α) and transforming growth factor beta (TGF)-β1 during the onset of liver fibrogenesis [43]. Interestingly, IOLE administration was significantly effective in reversing fibrosis in the liver. *Indigofera spps*. are used in ayurveda to treat several ailments including inflammation and cancer through their ability to suppress the transcription factor NF-κB [44]. Sethi et al. [45] reported that indirubin, an active ingredient from indigo, inhibits the invasion of cancer cells by suppressing MMP-9 upregulation.

In conclusion, this study has demonstrated that the extract of *Indigofera oblongifolia* leaves exhibits significant antioxidant, anti-fibrotic, and anti-apoptotic activities, and can reverse lead-induced oxidative damage in the liver through Nrf2/HO-1 pathway. These findings may be attributed to the manifold effects of the different polyphenols and flavonoids present in the extract.

## Supporting Information

S1 Fig The percentage of total polyphenols and flavonoids in the methanol extract of *Indigofera oblongifolia* leaves. Values are mean ± SEM of 3 measurements. Total polyphenols are expressed as % gallic acid equivalent per g dry extract. Flavonoids are expressed as % quercetin equivalents per g dry extract.
(TIF)

S1 Table Identification of phytochemical compounds by HPLC-ESI-MS in *Indigofera oblongifolia* leaves extract. (DOC)
