# Supplementary figures and images for: Indigofera oblongifolia Prevents Lead Acetate-Induced Hepatotoxicity, Oxidative Stress, Fibrosis and Apoptosis in Rats
Source: PLoS One. 2016 Jul 8;11(7):e0158965. doi: 10.1371/journal.pone.0158965 (PMC4938219; doi:10.1371/journal.pone.0158965)

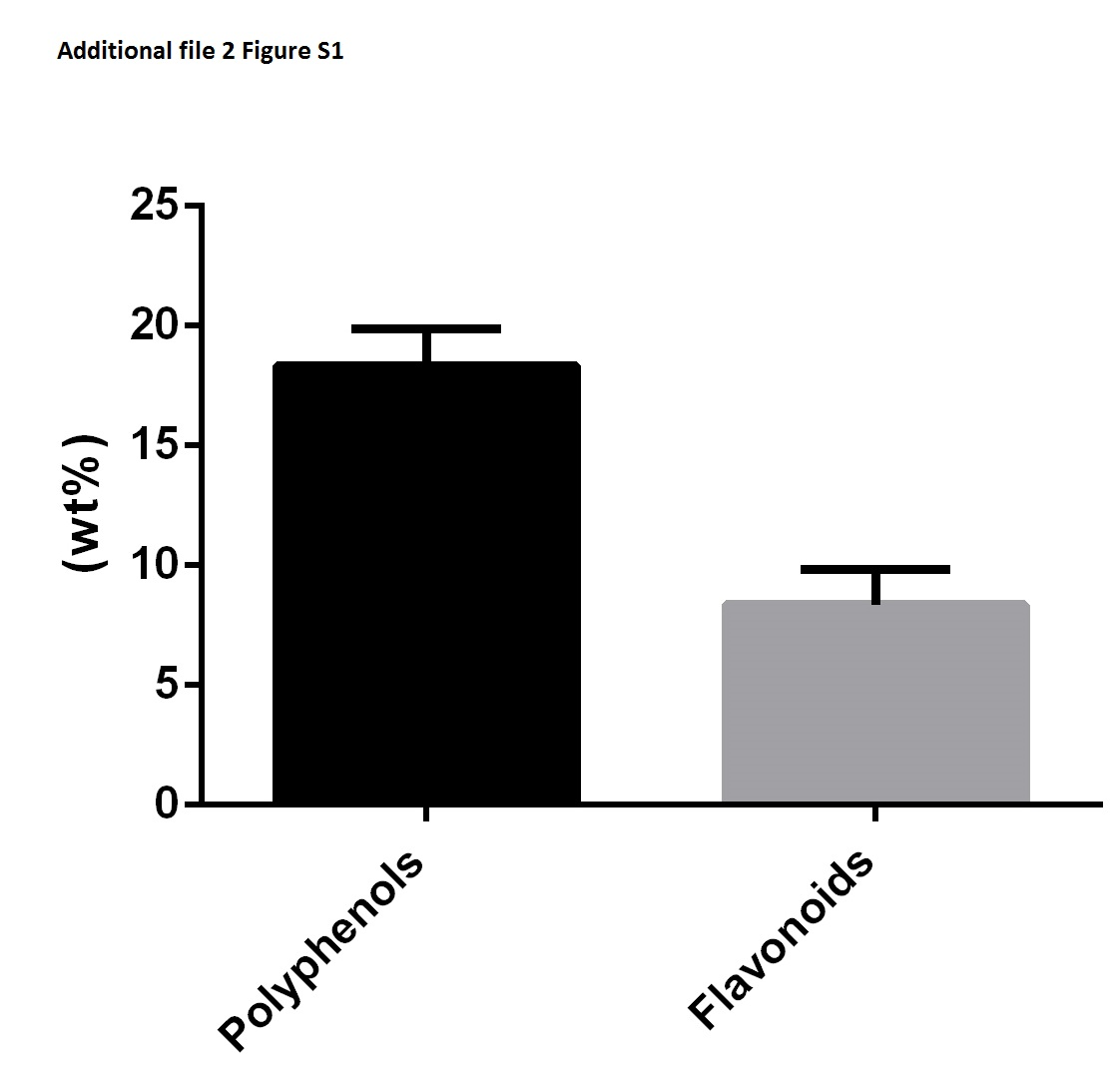

Supplement: S1 Fig — Values are mean ± SEM of 3 measurements. Total polyphenols are expressed as % gallic acid equivalent per g dry extract. Flavonoids are expressed as % quercetin equivalents per g dry extract. (TIF) [file pone.0158965.s001.tif]
