# Supplementary material for: Indigofera oblongifolia Prevents Lead Acetate-Induced Hepatotoxicity, Oxidative Stress, Fibrosis and Apoptosis in Rats
Source: PLoS One. 2016 Jul 8;11(7):e0158965. doi: 10.1371/journal.pone.0158965 (PMC4938219; doi:10.1371/journal.pone.0158965)
Supplement: S1 Table — (DOC) [file pone.0158965.s002.doc]

**S1 Table. Identification of phytochemical compounds by HPLC-ESI-MS in *Indigofera oblongifolia* leaves extract.**

|  | **Assignment** | **RT** | **[M – H]^−^**  **(m/z)** | **MS**  **(m/z)** |
| --- | --- | --- | --- | --- |
|  | Cyanidin 3-O-[2"-O-(2"'-O-(sinapoyl)xylosyl)glc]5-O-glc | 3.13 | 949 | 287, 449, 163 |
|  | 1-Methoxy indolyl glutathione | 9.48 | 469 | 368, 207, 162, 119 |
|  | Vanillic acid | 11.16 | 167 | 125, 152 |
|  | Apigenin 6-C-glucoside | 11.53 | 433.1 | 415, 397, 367, 337, 313, 283 |
|  | Caffeoyl putrescine | 12.12 | 251 | 161 |
|  | Cyanidin 3-O-[2"-O-xylosyl-6"-O-(p-Coumaroyl)glucoside]5-O-malonylglc | 14.66 | 975 | 287, 535, 727, 166 |
|  | Indigotin | 16.04 | 261 | 233, 217 |
|  | Quercetin mono-sinapoyl-di-O-[glc or gal] | 17.00 | 833 | 650, 493, 339, 163 |
|  | Luteolin 3,7′-di-O-glucoside | 17.31 | 447 | 285 |
|  | Lupinisoflavone | 17.79 | 353 | 179 |
|  | Apigenin-7-O-glucoside | 18.08 | 433 | 271, 191 |
|  | Indigo | 18.63 | 260.8 | 217 |
|  | 5-Benzoyloxypentyl glucosinolate | 19.06 | 510 | 190, 383, 433 |
|  | Quercetin- rhamnoside dimer 1 | 19.15 | 893 | 749, 603, 190 |
|  | luteolin C-glucoside C-xyloside | 19.55 | 581 | 485, 473, 461, 443, 425, 177 |
|  | Kaempferol 3-O-[rhamnosyl-Glucosylglucoside] 7-O-rhamnoside | 19.94 | 901 | 757, 741, 595, 516, 449, 433, 309, 287,177 |
|  | Luteolin | 20.16 | 285 | 243, 217, 199, 151, 133 |
|  | Luteolin C-6-(2" O-rhamnosyl)glucoside | 22.67 | 595 | 163, 337, 471 |
|  | Indol-3-ylmethyl glucosinolate | 24.14 | 449 | 327 |
|  | Indirubin, isomers | 26.10 | 261 | 233, 204, 103, 75 |
|  | 3'-O-methylluteolin 6-C-glucoside | 27.86 | 463 | 311, 163 |
|  | Methyl-O-quercetin rhamnosylglucoside | 34.30 | 625 | 537, 431, 259, 142 |
|  | Diferuloyl spermine | 47.20 | 555 | 353 |
|  | Diconiferyl alcohol glucoside | 47.61 | 521 | 431, 369, 269, 169 |
|  | β-Sitosterol glucoside | 49.26 | 577 | 447, 309, 264, 207,123 |
|  | Acylated (16:0) β-Sitosterol glucoside | 50.04 | 815 | 675, 619, 531, 494, 319, 123 |
|  | 1,2,2'-Trisinapoyl gentiobiose | 58.84 | 962 | 793.5, 709, 447, 123 |

Abbreviation: RT, retention time.
